# Unveiling Protein Functions through the Dynamics of the Interaction Network

**DOI:** 10.1371/journal.pone.0017679

**Published:** 2011-03-09

**Authors:** Irene Sendiña–Nadal, Yanay Ofran, Juan A. Almendral, Javier M. Buldú, Inmaculada Leyva, Daqing Li, Shlomo Havlin, Stefano Boccaletti

**Affiliations:** 1 Complex Systems Group, Universidad Rey Juan Carlos, Madrid, Spain; 2 Laboratory of Biological Networks, Centre for Biomedical Technology, Campus de Montegancedo, Madrid, Spain; 3 The Mina and Everard Goodman Faculty of Life Sciences, Bar-Ilan University, Ramat Gan, Israel; 4 Department of Physics, Minerva Center, Bar-Ilan University, Ramat Gan, Israel; 5 CNR- Istituto dei Sistemi Complessi, Sesto Fiorentino, Italy; University of Zaragoza, Spain

## Abstract

Protein interaction networks have become a tool to study biological processes, either for predicting molecular functions or for designing proper new drugs to regulate the main biological interactions. Furthermore, such networks are known to be organized in sub-networks of proteins contributing to the same cellular function. However, the protein function prediction is not accurate and each protein has traditionally been assigned to only one function by the network formalism. By considering the network of the physical interactions between proteins of the yeast together with a manual and single functional classification scheme, we introduce a method able to reveal important information on protein function, at both micro- and macro-scale. In particular, the inspection of the properties of oscillatory dynamics on top of the protein interaction network leads to the identification of misclassification problems in protein function assignments, as well as to unveil correct identification of protein functions. We also demonstrate that our approach can give a network representation of the meta-organization of biological processes by unraveling the interactions between different functional classes.

## Introduction

The rapid improvements in sequencing technologies are adding new sequences to the databases faster than the pace at which insights into their function could be gained. As a consequence, the vast majority of known genes and proteins have not been characterized experimentally, and their function is yet unknown [1]. Moreover, biological functions are not, in general, realized by individual proteins, but, rather, by networks of intricate interactions between numerous genes. The understanding of biological processes requires, therefore, a better knowledge of the functional organization of such networks. Indeed, the study of biological processes increasingly relies on the analysis of biological networks (BN), which has been used to tackle different levels of the functional organization of the cell. On the level of individual proteins, BN are often used to help to elucidate the molecular function of specific proteins [2], [3]. On the systems level, they are studied to reveal modules and functional sub-networks [4], [5].

An issue that has hardly been faced is that of the meta-organization of different functions in a single, integrated, network. Yook *et al.* [6] have concluded that most functional classes appear as segregated sub-networks of the full protein interaction network (PIN). Like most of the studies of BN, the results of Ref. [6] are based on parsing the static network, and do not allow the exploration of the meta-organization and the interactions of the sub-networks. We here, instead, give evidence that a dynamical approach to the analysis of BN based on their meta-organization not only enhances the prediction of the function of individual proteins, but also can reveal information on the network macro-scale of interactions between different biological functions.

As for predicting the function of individual proteins, two main strategies have been followed so far. The first relies on the analysis of the protein itself: e.g. its similarity to already annotated proteins, its structure, or its biophysical features [1], [7], [8]. The second one is, instead, based on high-throughput technologies providing data that may highlight the context in which the protein acts such as its sub-cellular localization, interactions with other proteins, and the conditions under which it is expressed (or the genes that are co-expressed with it) [2], [3]. High-throughput protein-protein interactions detection experiments allow nowadays a representation of the global cell functioning in terms of a network, with nodes representing proteins and edges representing the detected mutual interactions, with the goal of exploiting the properties of these networks for prediction purposes on the function of specific proteins. Notwithstanding the accomplishments of these analyses it is important to highlight that most high-throughput methods can suffer from high false positive and false negative rates [9] and, therefore, functional assignments that are based on these tools may lead to misclassifications.

Several past studies attempted already to determine to what extent the function of a protein depends on the way it is interacting with the others in the PIN. However, the use of such network representation for prediction requires the determination of the specific scale of the PIN that one has to consider for unveiling the individual protein's function. And, in this latter framework, the current state of the art includes, again, two types of approaches. From one side, several direct annotation schemes have been devised [10]–[14], with the common inspiration of analyzing the local scale features of the PIN, i.e. either basing the function prediction on the information that can be directly extracted from the protein neighborhood, or statistically assessing a probability for a protein to be assigned to a given function, depending on the actual number of its neighbors that are (or are not) pertinent to the same function. From the other side, more recent module assisted techniques [15]–[17] have attempted to make use of the extra knowledge arising from the meso-scale of clustered structures of the PIN, with first identifying dense agglomerates in the network that are loosely connected to other areas of the graph, and then to use this topological information for predictions on the protein specific function.

The approach we lay out constitutes a third, novel, strategy. We provide evidence that an alternative source of information is, in fact, the one arising from the analysis of how the modular PIN structure actually organizes the synchronization dynamics of an ensemble of oscillators. In particular, we show how the combination of synchronization features emerging in the PIN structure with a rudimentary classification of proteins based on expert manual assignment, allows, indeed, to gather information on misclassification problems, as well as to offer a more accurate function assignment that is consistent with more recent (and better refined) manual annotation of these proteins' function. Not less important is the ability of the approach we introduce to assess the coupling of different functional categories, to determine how closely associated they are, and which proteins participate in both of them.

## Materials and Methods

### Data

For our research we have used a typical and important network with rudimentary functional assignments derived from a *Saccharomyces cerevisiae* PIN, as reported in [18]. The data set is based on the work by von Mering *et al.* [9] who scored the reliability of 80,000 reported protein-protein interactions in the yeast. These were based on high-throughput interaction detection methods, such as *i)* yeast two-hybrid systems [19], [20], *ii)* protein complex purification techniques using mass spectrometry [21], [22], *iii)* correlated messenger RNA expression profiles [23], [24], *iv)* genetic interaction data [25], [26], and *v)* “in silico” interaction predictions derived from gene context analysis. From this set, Bu *et al.* [18] focused on 11,855 interactions (those featuring high and medium confidence levels) among 2,617 proteins. We here focus on the giant connected component of the PIN given in Bu *et al.* [18], consisting of 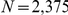 proteins and 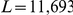 interactions.

As for the modular structure of the PIN, we initially refer to the partition in 13 functional categories given by the yeast protein catalog at the Munich Information Center for Protein Sequences (MIPS) [26]. Particularly, we use the data set in which each given protein is assigned to one of the functional categories (with proteins in multiple categories manually assigned by Bu *et al.* [18] to only one).

In order to test the validity of our findings, we will use the classification provided by the Gene Ontology consortium (GO) [27]. While MIPS attempts to provide a simple hierarchy with intuitive category structure that allows for manual browsing, GO aims at representing a fine granular description of proteins that provides annotation with a wealth of detailed information. Thus, MIPS gives a very rough division into a couple of dozens of categories and several hundreds of subcategories, whereas GO includes 29,983 different functional terms (as of March 2010). GO also provides a reduced version of its ontology (GOslim) that allows one to trace the detailed terms into more coarse-grained categories. In our analysis, we start with the single MIPS classification for each protein, and use the dynamical overlap method for identifying those proteins that are likely to be involved in more than one of the functional categories in our data (those ones forming the overlapping structures). As a validation, we refer to the classification of these proteins in GOslim, Namely, by manually mapping each GOslim term to one of the 13 MIPS categories, one is able to verify whether or not the assignment of the second function (provided by our method for each one of the proteins in the overlapping sets) is consistent with the functional annotation in GO.

### Dynamical Overlap Formalism

The method is based on the inspection of how oscillators organize in a modular network of dynamical interactions [28], by forming synchronization interfaces and overlapping communities [29], [30]. Here, we will consider a network of phase oscillators on top of the PIN. Thus, the transfer of function between neighboring proteins is performed through the synchronization of coupled oscillators. In order to explain how the method works, let us assume the PIN of the yeast is topologically divided into two main modules, 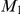 and 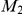, each one of them associated to a specific protein function. Every node (protein) in the network is an oscillator whose frequency 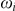 is set to 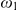 (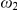) whenever the node 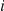 belongs to 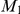 (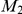), with 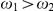. The phase dynamics of this network of 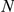 coupled oscillators can be described by
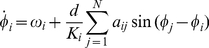
(1)where dot denotes temporal derivative, 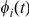 is the phase of the 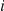-th oscillator, 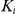 is the number of interactions that the 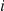-th protein has with the rest of proteins, 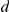 is some coupling strength, and 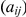 are the elements of the adjacency matrix representing the PIN [28], with 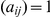 if there is an interaction between proteins 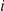 and 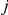, and 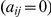 otherwise.

**Figure 1:**
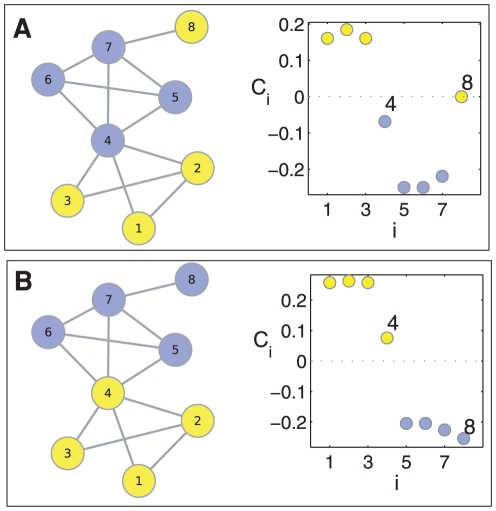
Graphical description of the dynamical overlap method. (**A**) A two module small graph composed of 8 nodes colored according to their membership to the functional module 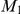 (yellow) or 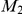 (blue), and corresponding 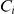 values after solving Eq. (1) with 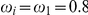 for 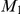 and 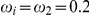 for 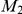. Nodes 4 and 8 have 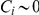 with this functional classification. (**B**) Same as in (**A**) but nodes 4 and 8 has been reassigned to modules 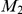 and 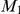 respectively. Now, node 8, behaves as a node truly from 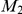 while node 4 behaves as an overlapping node between 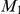 and 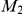 as 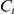 is again close to zero. All the network representations in this manuscript were produced with Cytoscape.

In the extreme case of fully separated modules the network dynamics would eventually (at large coupling strength 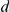) result in the clusters 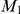 and 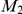 oscillating synchronously at a constant, different, frequency. If, however, there are just a few interactions between proteins of the two modules, the onset of a *synchronization interface* overlapping the two modules occurs, made of all those nodes displaying an instantaneous frequency that are actually oscillating in time around the mean value of the two frequencies characterizing the clusters [29]. The rest of nodes, out of the synchronization interface, oscillate at the frequency of the module they belong to. To quantify this behavior, we monitor the instantaneous frequency of each oscillator and we calculate the indicator 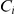,
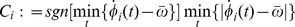
(2)which accounts for how close in time the frequency associated to protein 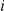 is to the average frequency 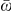 of the two clusters, 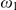 and 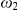. By fixing a confidence threshold 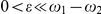, those proteins belonging to module 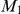 (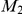) have 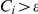 (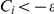) as they were assigned initially the frequency 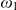 (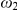), while 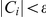 is the signature of a protein whose module membership is not clear, belonging to the synchronization interface between 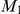 and 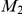. This behavior is graphically sketched in Fig. 1. There, a small graph composed of 8 nodes (Fig. 1A) clearly has two densely connected modules that do not coincide with the given functional classification denoted by the color of the nodes. Actually, node 8 does not have any link within its functional module, the yellow one, while node 4 is classified within the blue functional module but shares the same number of links with the other functional module. After solving Eq. (1) by assigning 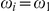 to nodes 1–3 and 8 (functional module 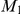), and 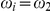 to nodes 4–7 (functional module 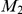), the corresponding 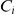 values extracted from Eq. (2) indicate that nodes 1–3 really belong to module 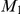 (as 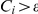), nodes 5–7 belong to module 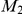 (as 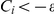), while nodes 4 and 8, whose 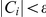, are the ones candidates to be overlapping between 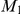 and 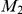. To solve this uncertainty, nodes 4 and 8 are reassigned to 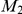 (blue) and 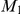 (yellow) respectively (Fig. 1B) and we observe that whereas 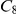 falls now within the area of module 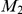, increasing the cohesion of the functional module, node 4 still lies within the synchronization interface (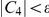) overlapping between both modules.

For the real situation of a PIN with 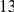 different functional modules (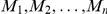), this can be done by integrating 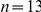 times the network dynamics described by Eq.(1). In each trial, the 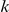-th module (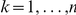) is assigned to the cluster frequency 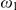, whereas the rest of the PIN is given the second cluster frequency 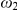, resulting in a series of 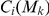 values. This time all those proteins initially assigned to 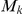 whose 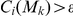, actually belong to module 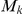, while if 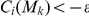 belong to another module different from 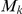. All those nodes whose 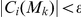 are labeled as belonging to the 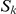 synchronization interface between module 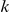 and the rest of the network. Then, a node is identified as an overlapping node between modules 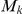 and 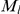 if, being a node from either 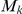 or 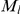, is in both 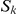 and 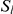, that is
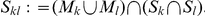
Finally, the set of nodes of module 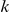 overlapping with module 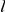, with 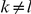, is
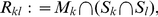
(3)which has two implications: *i)* while 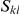 is symmetric in the indexes, 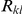 is not, and *ii)* 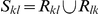 and, since 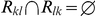, 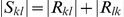.

Eventually, the degree of overlapping between two modules is then given by:
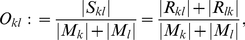
(4)which, therefore, provides a measure of how many nodes out of the clusters 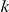 and 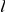 are forming the corresponding overlapping structure.

The main result of our method is, therefore, an index 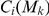 accounted by Eq.(2), that, for each protein 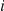, measures its degree of membership to module 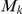 (i.e. a protein function). A value 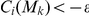 indicates that the protein exhibits a dynamical behavior different from that of the majority of proteins in 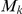, thus clearly belonging to other module. On the other hand, 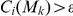 occurs when the protein performs as the rest of proteins assigned to the same module 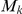, and this confirms that it is certainly member of 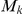. Finally, a value of 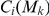 close to zero is the signature of a protein whose module membership requires further analysis as it could be the case of a protein belonging to two or more functional modules. Therefore, we are introducing an index that allows to check the accuracy of the initial functional assignment as well as predicting a second (or more) function of a protein.

## Results and Discussion

The application of the method given by Eq. (1) to the PIN and modular classification with 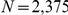, 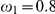, 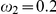, and 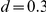, as described in the Materials and Methods section leads to 13 different series for 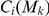 (being 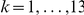 the functional module index and 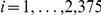 the protein index). In order to proceed with the full analysis of this data, we have to consider all possible combinations of these series to check whether a protein belongs to the functional module initially assigned or whether it is involved in more than one functional module. This can be done efficiently, as shown in the Figure S1, but, to illustrate the principles underlying the method, we will just focus on a single pair of functions.

**Figure 2:**
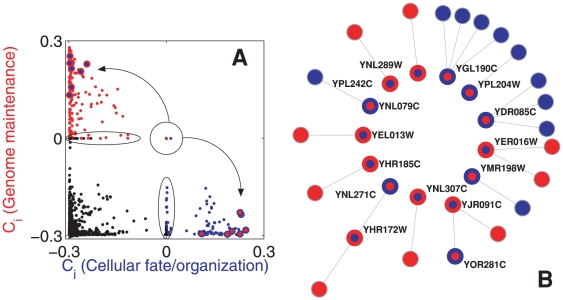
Identification of misclassified proteins. (**A**) 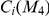 and 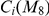 values for all proteins in the PIN of the yeast. The color indicates the functional module initially assigned to each protein (blue for 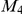, red for 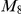 and black for the rest). The method identifies 15 proteins (within the circle) with a twofold assignation (the initial and the predicted one). After re-assignation to the predicted function, the new 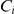 values of the 15 proteins are depicted as circles bordered with the color of that function, and lie together with those other proteins of the same function, indicating an original misclassification. (**B**) Visualization of the network backbone, made of the 15 misclassified proteins and their neighbors. Same color code as for (**A**).

Figure 2A shows the values of the indexes 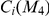 and 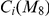, being 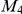 and 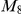 the *Cellular fate/organization* and *Genome maintenance* functional modules. We plot proteins initially assigned to 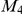 (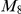) in blue (red), while the rest of proteins are plotted in black. Notice that most of the black points are concentrated around 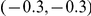, as the corresponding proteins neither belong to 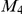 nor 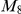. The majority of proteins in 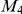 (blue) and 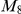 (red) are located close to 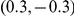 and 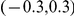, respectively. The blue points inside the ellipse correspond to proteins initially classified as 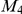 that are not belonging to 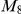 (as 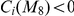), but for the very same to 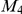 is under question (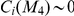). When examining the indexes for the rest of modules, one finds out 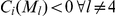. Therefore, we infer that these proteins do, indeed, belong to 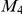 although weakly. The same arguments apply for the red points lying within the other ellipse: they are proteins weakly ascribed to 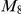. A completely different situation is that of those points distributed around 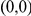 (inside the circle, mostly of the points superimposed). They correspond to 15 proteins whose unique membership to 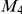 and 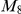 cannot be asserted. When checking the rest of 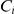 values, one finds that none of these proteins can be assigned to modules other than 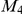 and 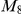, thus again they are weakly associated to both functions 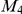 and 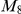 (one of them being the initially assigned function, and the other the predicted one). The novelty here is that there is a twofold assignation, which could be considered as the trace of multi-functional proteins.

Before claiming for multi-functionality, it is mandatory to check if such a multi-assignment holds when the initial modular structure changes. This is tantamount to reassign each one of these proteins to the predicted function and check whether the corresponding protein is still located around 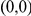, otherwise the multi-functionality is simply an artifact. The new 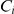 values for the 15 proteins (after reclassification) are shown in Fig. 2A as circles bordered with the color of the predicted function. The remarkable result is that the emerging dynamics behavior agrees with the new classification, as the 15 proteins are no longer overlapping and move now to the areas corresponding to the predicted function. If we take into account the number of connections a given protein is forming with elements belonging to any one of the other modules in the graph, 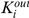, the emerging dynamics is reflecting the fact that the original and predicted assignments correspond, respectively to 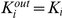 and 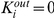, that is, the predicted classification makes the functional module more cohesive (see Fig. S2B). For the sake of visualization, Fig. 2B shows the backbone of the original PIN made of the 15 proteins and all their neighboring proteins. While the original function assignment classified the proteins in modules in which they do not have physical interactions, the reclassification is able to unveil the participation of the proteins to the correct module. For example, according to GO, YHR172W is not involved in Cellular fate/organization but in Genome maintenance (see Table S1), which is in agreement with the classification pointed by our method.

Notice that, in the full analysis, the number of proteins featuring an overlapping behavior is 418 (see the full list 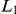 in Table S1 and Fig. S2) out of which 103 proteins have no functional annotation in GO and 200 had two or more different function annotations in GOslim. For these latter ones, a comparison with the functions assigned by GO reveals that in 87 cases the predicted function is in agreement with one of the GO assignments. The expected average number of matching of the proteins in 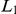 for a random function assignment is 25. The p-value for the significance of this result is 0.0001, and it can be established by performing 1,000 random reshuffles of function assignment, and verifying the average number of matches (which in this case was 25). The highest number of random matches was 50 (in 1/1,000 cases), well below the observed 87. As a result, one can claim an original misclassification and, consequently, the method can be used to cure errors in a given protein function classification.

**Figure 3:**
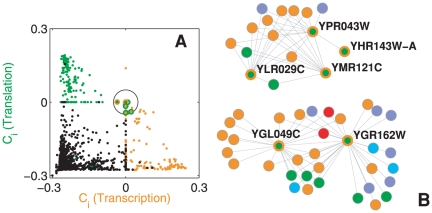
Identification of multi-functional proteins. (**A**) 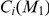 and 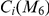 values for the 2,049 proteins in the PIN of the yeast after curation. The color indicates the functional module initially assigned to each protein (orange for 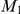, green for 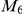 and black for the rest). The 30 proteins located inside the circle remain there after re-assignation to the predicted function, and are depicted as circles bordered with the color of that function. (**B**) Visualization of the network backbone made of 6 (out of 30) of the multi-functional proteins in (**A**).

With the guidance of the information obtained so far, we have reclassified all proteins of 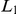 to the corresponding predicted functions, and extracted the subgraph of the original PIN for which each functional module corresponds to a connected component (i.e. we pruned out all those other proteins that were assigned a given function in the MIPS classification, but did not have any interaction with other elements of the same function). The result is a new interaction network made of 2,049 nodes and 9,941 links, that we take for a new set of numerical trials, resulting in a second list 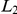 of 211 potentially multi-functional proteins (reported in Table S2). The situation, is now radically different: at variance with the results of Fig. 2, Fig. 3 shows that the multi-functional nature of the 30 proteins inside the circle (the subset of 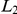 obtained when comparing 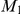 (*Transcription*) and 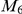 (*Translation*), is indeed genuine, as the final outcome does not depend on whether the proteins are classified according to the assigned or predicted functional modules (see Fig. S3A). This is further confirmed by the simultaneous reclassification of each one of the proteins of 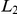 into the predicted function, and by monitoring the change in the out-degree, 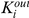, calculated with the predicted and the original classification (shown in Fig. S3B).

**Figure 4:**
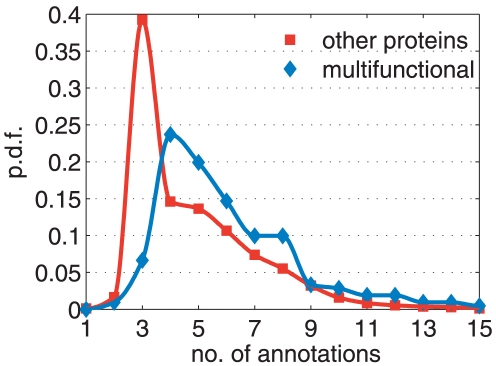
Statistical assessment of protein multi-functionality. Probability density function of the number of different GO annotations (see Materials and Methods section) of the 211 overlapping proteins in 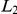 (blue diamonds), as compared to the probability of other proteins in the rest of the data (red squares). Continuous lines are shape-preserving interpolations.

An independent test of the validity of that assignment is to assess the multi-functionality character of the proteins in 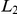 by comparison with the more accurate GO classification scheme. One can count the number of different GO annotations for each of the proteins in 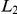, and the corresponding distribution of multiple assignments in the rest of the data. The difference between the two distributions (see Figure 4) is significant (p-value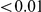, as for conventional t-test). Namely, the average number of different function assignments in 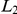 is 6.7, with mode 4, while in the other proteins one finds 4.9 and 3 respectively. Moreover, the standard deviation of the distribution of functions in 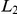 is significantly greater than that of the other proteins. This confirms that the proteins in 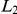 come from a population with higher multi-functionality with respect to the population of other proteins.

**Figure 5:**
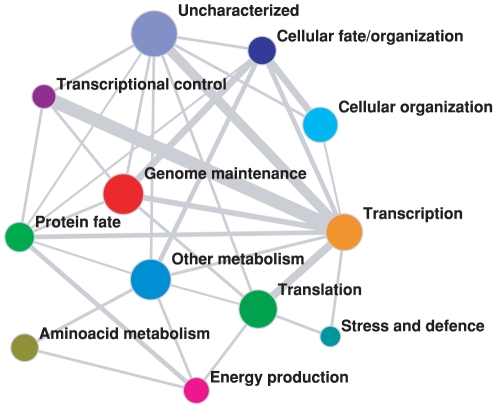
Coarse grained representation of the PIN in terms of cell functioning and coordination. The size of nodes is proportional to the total number of proteins participating to the corresponding function, the width of the links is proportional to the size of the corresponding overlapping interface. The full picture of the structure of these overlaps is reported in the Table S3.

Finally, the method allows also to assess a coarse-grain representation of the PIN, showing the way each biological function is interacting with the others. In Figure 5, each specific cell function is represented by a node whose size is proportional to the total number of proteins participating in that function. The width of each link is proportional to the number of multi-functional proteins provided by our method (Equation (4)). The resulting network representation of the full cell functioning suggests numerous insights about the organization and control of biological functions. As one might expect, there is a strong link between Transcription, Translation and Transcriptional control. But these functions have almost no common proteins to functions like Genome maintenance, Cellular organization or Metabolism. Interestingly, the results show that there are no shared proteins between Amino-acid metabolism and Protein fate, suggesting that even though these two processes may seem related there are no known common mechanisms that control both functions.

We have then given evidence that a proper inspection on the meso-scale interactions of a generated network of dynamical systems can provide useful information on the micro- and macro-scale processes through which biological processes are organized in a cell. The method is not only able to predict and reassign the function of a given protein, but also to describe qualitatively the main functional interactions that lead to the global functioning of the organism. It is worth highlighting that the present application only focused on unveiling proteins with double functionality, while the method can be easily applied to gather information also on proteins bridging among more than two different biological functions (such an evidence will be reported elsewhere). The core of the presented results gives insights on how molecular functions are networking at different scales, as well as on how to design (or engineer) proper drugs, or mechanisms to control (or regulate) the biological interactions responsible for the functioning, or malfunctioning, of a cell.

## Supporting Information

Figure S1
**Identification of misclassified proteins.** The proposed tool is providing the behavior of each protein in the PIN through the indicator 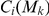, that crucially depends on its original functional classification. Each panel corresponds to the competition trial between module 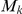 at frequency 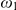 (in black symbols) and the rest of modules 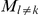 at frequency 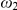 (in different symbols and colors). The size of each module is written between brackets. Nodes belonging to the corresponding synchronization interface (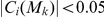, gray band) are marked in full face. Those nodes corresponding to overlapping proteins (those appearing in two synchronization interfaces, 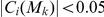 and 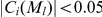) are encircled with the color of the corresponding overlapping function. Parameters used in Equation (1): 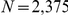, 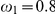, 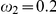 and 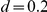 (**A**), 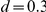 (**B**). 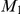 (Transcription), 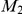 (Other metabolism), 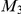 (Uncharacterized), 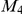 (Cellular fate/organization), 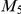 (Protein fate), 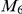 (Translation), 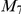 (Amino-acid metabolism), 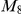 (Genome maintenance), 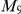 (Cellular organization), 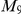 (Energy production), 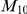 (Stress and defence), 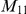 (Transcriptional control), 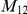 (Transport and sensing), and 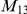 (Transport and sensing).(EPS)

Figure S2
**Identification of misclassified proteins.** (**A**) Dynamical behavior of the 418 overlapping nodes. In blue when the modules are defined according to the original classification (MIPS). Given that the overlapping node 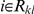 is simultaneously in 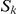 and 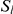, we represent with a circle its 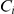 value in 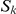 and with a square its 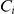 value in 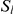. In red we represent the same values as before but when the modules are modified to take into account the function predicted by our method for the overlapping nodes. Same parameters as in Fig. S1B. (**B**) Topological behavior. 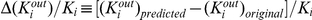, change in the ratio between out-degree (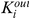, number of connections a given protein is forming with elements belonging to any one of the other modules in the graph, and the underscores predicted/original stay for the calculation of 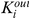 in the corresponding annotation) and total degree (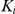, degree of the protein, independent on the specific classification of the protein) of the proteins in 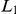 (green dots) and the rest of the proteins (black dots) when reassigning the function given by MIPS to the predicted one. The results show that, while all non overlapping proteins (black points) are grouped around 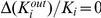 (i.e. they do not substantially change their *in-out* connections due to the change in the classification of the overlapping proteins), the members of 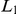 (green points) appear grouped around 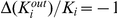, thus reflecting the fact that the original and predicted assignments correspond, respectively to 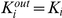 and 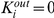. This indicates that in the original classification of the proteins in 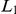 they did not have interactions with other elements of the original functional module, whereas the predicted classification assigns them to the proper functional class.(EPS)

Figure S3
**Identification of multi-functional proteins.** (**A**) Dynamical behavior of the new set 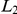 of overlapping proteins. In blue, 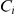 values of the set of overlapping proteins between modules 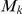 and 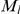 with the new cured classification (same as in Fig. 3). As in Fig. S2, we plot the 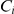 value of the overlapping node 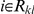 with circles when is in 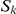 and with squares when in 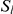. In red we represent the same values as before but when the modules are modified to take into account the function predicted by our method for the overlapping nodes. (**B**) Topological properties of the cured PIN. Change in the ratio between out-degree (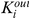) and total degree (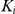) of the proteins in 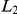 (green dots) and the rest of the proteins (black dots) when reassigning the function given by MIPS to the predicted one. Parameters used in Eq. (1): 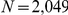, 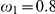, 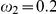 and 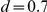.(EPS)

Table S1
**List** 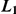 **of proteins.** Full list 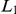 with the 418 overlapping proteins resulting from the first iteration of the dynamical overlap method for the PIN of the yeast (see Materials and Methods and Fig. 2). For each protein, we provide the OLN (Ordered Locus Names), the MIPS classification, whether or not this function is annotated in GOslim, the predicted function and whether or not this predicted function is also provided by GOslim. The first 87 proteins correspond to cases in which the predicted function is in agreement with one of the GO assignments.(PS)

Table S2
**List** 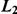 **of proteins.** Full list 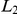 with the 211 overlapping proteins resulting from the second iteration of the dynamical overlap method for the curated PIN of the yeast (see Text and Fig. 3). The curation of the PIN consists in exchanging the annotated function by MIPS of the 418 proteins from 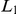 with the function predicted by the overlap and removing those proteins that become isolated within the functional module. Again, for each protein, we provide the OLN (Ordered Locus Names), the MIPS classification and the predicted function.(PS)

Table S3
**Multifunctional distribution of proteins in** 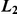 **.** 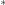
Module index. 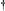
Number of proteins within the 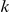-module. 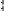
Overlapping nodes belonging to 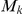. 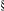
Number of proteins belonging to the 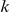-module overlapping with module 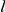.(PS)
